# Asphyxia due to laryngeal spasm as a severe complication of awake deep brain stimulation for Parkinson’s disease: a case report

**DOI:** 10.1186/s12883-016-0736-7

**Published:** 2016-11-08

**Authors:** Kajetan L. von Eckardstein, Friederike Sixel-Döring, Stephan Kazmaier, Claudia Trenkwalder, Jason M. Hoover, Veit Rohde

**Affiliations:** 1 Department of Neurosurgery, Universitätsmedizin Göttingen, Robert-Koch-Straße 40, 37075 Göttingen, Germany; 2 Department of Anasthesiology, Universitätsmedizin Göttingen, Göttingen, Germany; 3 Paracelsus Elena-Klinik, Kassel, Germany; 4 The Texas Brain and Spine Institute, Bryan, TX USA

**Keywords:** Asphyxia, Complication, Deep brain stimulation, Parkinson’s disease, Laryngeal spams

## Abstract

**Background:**

In accordance with German neurosurgical and neurological consensus recommendations, lead placements for deep brain stimulation (DBS) in patients with Parkinson’s disease (PD) are usually performed with the patient awake and in “medication off” state. This allows for optimal lead position adjustment according to the clinical response to intraoperative test stimulation. However, exacerbation of Parkinsonian symptoms after withdrawal of dopaminergic medication may endanger the patient by inducing severe “off” state motor phenomena. In particular, this can be a problem in awake craniotomies utilizing intraoperative airway management and resuscitation.

**Case presentation:**

We report the case of a PD patient with progressive orofacial and neck muscle dystonia resulting in laryngeal spasm during DBS lead placement. This led to upper airway compromise and asphyxia, requiring resuscitation.

**Conclusions:**

Laryngeal spasms may occur as a rare “off” state motor complication in patients with PD. Other potential causes of intraoperative difficulties breathing include bilateral vocal cord palsy, positional asphyxia, and silent aspiration. In our practice, we have adjusted our medication regimen and now allow patients to receive their standard dopaminergic medication until the morning of surgery. Neurologists and neurosurgeons performing lead placement procedures for PD should be aware of this rare but unsafe condition to most optimized treatment.

## Background

Deep brain stimulation (DBS) of the subthalamic nucleus (STN) is indicated for patients with severe motor symptoms in advanced Parkinson’s disease (PD) [1]. In accordance with current consensus recommendations, the majority of neurologists and neurosurgeons prefer awake craniotomies [2, 3]; although some groups [4] perform this surgery under general anesthesia. Awake surgery ensures optimal lead placement rather than relying solely on anatomical landmarks and electrophysiological data gained by microelectrode recordings [5]. The effect of intraoperative macrostimulation on PD symptoms should correspond to the patient’s preoperative response to levodopa. Furthermore, intraoperative test stimulation with the patient awake allows assessment of potential stimulation-associated side effects with consecutive adaption of lead position. In preparation for surgery, dopaminergic medication is usually withdrawn approximately twelve hours before surgery. Thus, patients are in their “medication off” state when entering the operating theater. “Off” state PD symptoms may be exacerbated by exhaustion.

In DBS patients undergoing awake lead placement, loss of airway is critical, as the patient’s head is placed in the stereotactic frame, which is attached to the operating table.

In this case report we highlight the rare complication of severe intraoperative laryngeal spasm with secondary asphyxia in a patient with PD during DBS placement, likely due to withdrawal of dopaminergic medication.

## Case presentation

The patient is a 59-year-old man diagnosed with PD at the age of 49 years. Fluctuations with recurring “off” states and peak dose dyskinesias had severely diminished the patient’s quality of life. Thus, the patient was deemed a candidate for bilateral subthalamic DBS. Multidisciplinary evaluation with 75 % positive response in the standardized levodopa test, exclusion of cognitive decline or psychiatric comorbitity, and absence of structural brain damage potentially relevant to the lead placement led to the unanimous decision for DBS placement. When decision was made to proceed to surgery the medication consisted of 1112.5 mg of levodopa and 150 mg of piribedil. There was no history of orofacial or cervical dystonia.

In the “on” state the patient was alert without any signs of psychiatric comorbidity. Neuropsychological testing was adequate. No cranial nerve deficits were found and swallowing was normal. Gait and postural stability was normal with minimal rigidity in the neck and right arm. Coordinative motor skills such as rapid pro- and supination were restricted in the left hand. Dyskinesias were evident with moderate impairment. In the “off” state after the medication was held for 12 h, the patient showed mild dysarthria and dysphonia, resting tremor of the right arm and leg, as well as slight action tremor in the right hand. Rigidity was severe in the neck, marked in the right, and mild on the left side. Motor skills of the hands were markedly impaired. Gait was slow but unaided and comprised of by intermittent freezing; there was mild dystonic posturing of the right foot. There were no signs of laryngeal spasms, however.

On the day before surgery, the last dose of dopaminergic medication was administered at 07:00 p.m., the bedtime dose of levodopa was held to facilitate intraoperative testing. On the day of the operation, the patient underwent placement of the stereotactic frame under local anaesthesia around 09:00 a.m. After planning standard STN coordinates and trajectories, the patient was placed in a semi-sitting position on the O.R. table, with the frame attached to the table. At the patient’s request, the head was slightly flexed anteriorly for comfort. The left electrode was placed uneventfully. When performing the bur hole on the right side, the patient complained of cramping in the neck and facial muscles as well as difficulties breathing although at that point, pulse oximetry showed good saturation readings at ≥95 %. He progressed to dystonic dysarthria [6]. Microelectrode recordings had already been done and macroelectrode test stimulation was about to begin, when the patient showed high-pitched inspiratory stridor. Pulse oximetry showed decreasing oxygen saturation at 02:15 p.m. and, shortly thereafter, narrow complex tachycardia was noted. Within a minute, the patient became unresponsive. Cardiac resuscitation was initiated and the patient was fiberoptically intubated after removal of the front bar of the stereotactic frame. During fiberoptic intubation laryngeal spasm was confirmed visually. A transthoracic echocardiogram obtained immediately after successful resuscitation showed no cardiac pathology or any air bubbles. Stimulation using the implanted lead to resolve the symptoms was not possible as at that time the lead was subcutaneously tunnelled with no impulse generator attached. The right permanent electrode was placed without further testing according to microelectrode recordings. 50 mg levodopa was administered over a nasogastric tube every two hours during the subsequent postoperative period. Postoperative head CT scan done immediately after the procedure was normal. The patient was transferred to the intensive care unit and was extubated at 02:00 p.m. the following day without any neurological deficit or signs of laryngeal spasms. By then, the nasogastric tube was discontinued and the preoperative medication was resumed. Cardiac workup was negative. The impulse generator was implanted 7 days later and the patient showed good symptom control of PD.

## Conclusion

The aforementioned complication has not been described before. We suspect laryngeal spasms developed in the course of the dystonic symptoms due to the lack of dopaminergic stimulation as a cause.

Throughout the literature, the terms “laryngeal dystonia” and “laryngeal spasm” are occasionally used synonymously, especially in the context of underlying neurological disease, although laryngospasms can also be elicited by local inflammatory reactions, regardless of a dystonic disorder. Both conditions were associated with PD and other neurological conditions, such as multiple system atrophy, and can subsequently lead to upper airway obstruction [7].

Effects of PD on respiratory function are well-known. Dyspnea as a symptom in PD was first described in 1958 [8]. Without naming the likely diagnosis of laryngeal spasm due to a laryngeal dystonia, levodopa-responsive upper airway obstruction was characterized further in 1989. Featuring flow-volume loops on a patient with PD, the author clearly demonstrated a significant airway compromise in the “medication off” state, with improvement of forced vital capacity after administration of levodopa [9]. Further clinical cases can be found in the literature. One of these reports describes the case of a 71-year-old man with PD undergoing elective bowel surgery. He developed upper airway obstruction requiring re-intubation after discontinuation of his Parkinson medication [10]. The second case was a 61-year-old PD patient also required re-intubation for laryngospasm after uneventful elective abdominal surgery [11]. In both cases, dopaminergic medication was resumed thereafter and symptoms completely resolved.

Other possible explanations for upper airway compromise in PD patients include rarely laryngeal spasms induced by neuroleptics and the well known symptom of silent aspiration [12–15]. It seems unlikely, however, that silent aspiration played a role in the pathogenesis of rapidly progressive difficulties breathing and secondary asphyxia, as seen in our patient.

A recent forensic publication draws some attention to the aspect of positional asphyxia in predisposed patients. In a retrospective analysis of patients with positional asphyxia being the cause of death, the authors report on a patient with severe PD and progressive supranuclear palsy and clinical disability to extend her head by her own, leading to difficulties breathing in the flexed position that she was found in [16]. Although highly unlikely, antecollis head position in the semi-sitting position with the frame rigidly attached to the operating table should be within the scope of possible attributing factors of breathing difficulties in awake surgery.

Furthermore, we have considered a stimulation induced dystonic reaction upon lead insertion and test stimulation on the left side that gradually developed over the course of the remaining procedure. However, after reviewing the macrostimulation protocol of the left side, we did not experience any dystonic reaction, neither upon electrode placement nor stimulation. Hence, we do not believe, that any effect will be elicited with a detached stimulator thereafter.

We hypothesize that upper airway obstruction due to “off” state induced laryngeal spasm eventually necessitated resuscitation in our patient. Levodopa withdrawal of 12 h in the context of preoperative evaluation was likely not long enough to result in a clinically relevant laryngeal spasm. However, as the prolonged levodopa withdrawal of over 19 h may enhance exhaustion and thus severity of “off” state symptoms by impaired sleep the night before, we propose to allow levodopa medication until six hours before surgery. This may help avoid the very severe “off” state this patient experienced. So far, we have not had any difficulties with clinical testing during surgery, as clinical symptoms were fully present during time of macrostimulation. Any change of voice or breathing pattern during DBS procedures should alert neurologist, neurosurgeons, and anaesthesiologists to consider life-threatening laryngeal spasm. We now utilize a neutral position of the head when attaching the stereotactic frame to the operating table. This has become important in patients that prefer to have the head of bed somewhat higher as sliding down in the course of the operation will increase flexion of the head.

## Abbreviations

DBS: Deep brain stimulation
PD: Parkinson’s disease
STN: Subthalamic nucleus
